# Breast Cancer Detection with Quanvolutional Neural Networks

**DOI:** 10.3390/e26080630

**Published:** 2024-07-26

**Authors:** Nadine Matondo-Mvula, Khaled Elleithy

**Affiliations:** Department of Computer Science and Engineering, University of Bridgeport, Bridgeport, CT 06604, USA; elleithy@bridgeport.edu

**Keywords:** quanvolutional neural network, quantum convolutional neural network, quantum machine learning, breast cancer, quantum neural network

## Abstract

Quantum machine learning holds the potential to revolutionize cancer treatment and diagnostic imaging by uncovering complex patterns beyond the reach of classical methods. This study explores the effectiveness of quantum convolutional layers in classifying ultrasound breast images for cancer detection. By encoding classical data into quantum states through angle embedding and employing a robustly entangled 9-qubit circuit design with an SU(4) gate, we developed a Quantum Convolutional Neural Network (QCNN) and compared it to a classical CNN of similar architecture. Our QCNN model, leveraging two quantum circuits as convolutional layers, achieved an impressive peak training accuracy of 76.66% and a validation accuracy of 87.17% at a learning rate of 1 × 10^−2^. In contrast, the classical CNN model attained a training accuracy of 77.52% and a validation accuracy of 83.33%. These compelling results highlight the potential of quantum circuits to serve as effective convolutional layers for feature extraction in image classification, especially with small datasets.

## 1. Introduction

The National Library of Medicine (NIH) reports that breast cancer is the second most common cancer in women after skin cancer with 1 in 10 new cancer diagnoses each year. In general, there are two types of breast cancer tumors: non-cancerous (benign) and cancerous (malignant). Cancerous breast tumors occur when malignant cells form in tissues of the breast with first signs of a lump or change in the breast. When the tumor is diagnosed as malignant the physician will perform a biopsy to determine the aggressiveness of the tumor. According to estimates from the National Breast Cancer Foundation, Inc., in 2023 about 30% of all new female cancer diagnoses will be breast cancer and 43,700 women will die from breast cancer in the US. Although this has decreased since 1989 because of early cancer prevention efforts, breast cancer persists as one of the most common cancers in women. In general, breast cancer often happens due to changes in the genetic material (DNA) or with an increase in age. For these reasons, breast cancer screening such as mammogram (X-ray of the breast), Breast Magnetic Resonance Imaging (MRI), clinical breast exam, etc., is recommended before there are signs of the disease. Nevertheless, false positive test results caused by a misdiagnosis by a physician may lead to more tests which is expensive and time-consuming. Additionally, false negative test results occur when mammograms miss some cancers which in turn delays cancer detection and subsequent timely commencement of treatment. Further, Computer-Aided Detection (CAD) systems, first approved in 1998 by the Food and Drug Administration (FDA), have been developed to improve and therefore to reduce the number of false-negative interpretation of mammographic detection of breast cancer at screening [1]. Although image-processing techniques have advanced dramatically in recent years, CAD systems still use conventional processing methods and have been limited in their ability [2].

Artificial intelligence (AI), especially machine learning models, have been proven to be successful in early detection and treatment of various types of cancer, in particular breast cancer. Further, machine learning (ML) models have been used with traditional computers to interpret CT scans, mammograms and aid eliminating diagnostic errors, thus increasing the survival chance of patients [3]. Deep learning (DL) is a subset of machine learning and employs the techniques of sending an image dataset through several layers of artificial neural networks to learn complex patterns. Researchers have proposed algorithms with evolutionary changes in cancer research using cancer images from different modalities along with detecting cancer cells, cancer type classification, lesion segmentation, etc., [4,5,6]. The intersection of quantum and machine learning may exponentially increase image details because of its usage of quantum mechanics. This in turn results in an acceleration of the process because quantum computers can act simultaneously on multiple values (quantum superposition). Quantum machine learning models show potential in problems where data are limited and some of the models show polynomial speedup in contrast to their classical counterparts. In cancer research, quantum machine learning may help with improved therapies for cancer treatment and cancer diagnostic imaging by discovering complex patterns that are not readily detectible by trained medical specialists, consequently decreasing the number of false positives [7]. However, quantum machine learning models are mostly theoretical proposals at this stage, due to limited quantum computers and their susceptibility to error and hence only accessible to scientists and qualified specialists. Many researchers have proposed solutions with promising results. For instance, the authors in [8] developed a hybrid quantum neural network model for drug response prediction in cancer patients. This resulted in 15% greater effectiveness than its classical counterpart. Vashisth et al. [9] use a quantum version of the support vector machine (SVM) model, namely quantum SVM, to classify malignant breast cancer diagnosis to guarantee an exponential speedup over its conventional model. In [10], the authors used a Quantum Boltzmann machine (QBM) running on a quantum annealer to predict if non-small cell lung cancer patients have adenocarcinoma or squamous cell carcinoma. The model achieved an accuracy of 95.24%.

The primary contribution of this research lies in implementing quantum convolutional layers as feature extractors in a neural network model to capture robust features from ultrasound breast images for breast cancer detection. Our proposed method utilizes angle encoding for data mapping and employs quantum circuits with a fixed sequence of parameterized gates as quantum convolutional layers with a 3 × 3 kernel size. This enables the transformation of spatially local subsections of input data, facilitating training on extensive, high-dimensional datasets. Additionally, we have chosen to train our model on a subset of the dataset, aligning with our hypothesis that Quantum Neural Networks (QNNs) can perform effectively even with limited data, addressing a common challenge in medical imaging.

In our research, we encountered a significant limitation: unitary operations and measurements are computationally demanding, leading to increased computational load and training time. To expedite the training process and demonstrate the ability of feature extraction using only quantum convolutional layers, we reduced the original image size from 28 × 28 to 14 × 14. Furthermore, our implementation utilizes two circuits, each comprising 9 qubits, effectively representing a 3 × 3 kernel designed to traverse an image of 14 × 14 pixels.

Our calculations suggest that processing a single image with this setup would require approximately 72 h, accounting for the minimum 30-min waiting time per job on a real quantum computer. Considering the scale of our experiment, which includes 546 training images and 78 validation images, even when only a subset is used for the training, it is clear that executing the experiment on a real quantum computer presents significant challenges. Each epoch, with a batch size of 10, would require 54 days to complete, making the feasibility of this approach questionable. Therefore, we have decided to conduct our experiment using a quantum simulator. The rest of the paper is structured as follows: Section 2 gives an overview of Classical and Quantum Neural Network, Section 3 discusses related work, Section 4 describes the proposed methodology, and Section 5 discusses the result of the study. Finally, Section 6 presents the discussion and concluding remarks are given in Section 7.

## 2. Classical and Quantum Neural Network

### 2.1. Quantum Computing

Quantum Computing (QC) is a multidisciplinary field combining the ideas of classical information theory, computer science, and quantum physics. With quantum physical phenomena, such as quantum superposition (the ability of a quantum system to be in multiple states at the same time) and quantum entanglement (the allowance of reliable transmission of quantum states (teleportation), it is believed that quantum computers offer speed advantage over classical computers and have the potential to solve complex problems [11].

The fundamental concept of a quantum computation is quantum bit (qubit), analogous to the classical bit. A qubit can be in the states in Dirac notation, $\left|\left.0\right\rangle \right.$ and $\left|\left.1\right\rangle \right.$, which is a vector in a two-dimensional complex vector space. Further, a qubit can also be in a state other than $\left|\left.0\right\rangle \right.$ or $\left|\left.1\right\rangle \right.$. In this case, the qubit is in a superposition, a linear combination of states:

(1) $$\left|\left.\psi \right\rangle \right.=\left.\alpha \right|\left.0\right\rangle +\left.\beta \right|\left.1\right\rangle$$

α and β are complex numbers and the states $\left|\left.0\right\rangle =\left[\begin{array}{c} 1\\ 0 \end{array}\right]\right.$ and $\left|\left.1\right\rangle =\left[\begin{array}{c} 0\\ 1 \end{array}\right]\right.$ are known as computational basis states and form an orthonormal basis for a vector space. The measurement of a quantum state produce either the result 0 with probability of ${\left|\alpha \right|}^{2}$ or the result 1 with a probability of ${\left|\beta \right|}^{2}$. In the case of an equal superposition, both probabilities are 50% = 0.5. In all cases, these probabilities must sum up to 1 (${\left|\alpha \right|}^{2}+$ ${\left|\beta \right|}^{2}$ = 1). Furthermore, the state of a quantum system can be manipulated through one-qubit gates, two-qubit gates, and multiple-qubit gates which are unitary (U) matrix transformations on a quantum system with the following requirements:

(2) $$UU^{†}=U^{†}U=I$$

where $U^{†}$ is the conjugate transpose of the U and I is the identity matrix [12]. Frequently used gates are Pauli Spin matrices (relevant for making flipping states or making rotations in a qubit), Hadamard matrix (responsible for the superposition of qubits), and phase matrix defined as follows:

$$Pauli\;X=\left[\begin{array}{cc} 0 & 1\\ 1 & 0 \end{array}\right]$$

$$Pauli\;Y=\left[\begin{array}{cc} 0 & -i\\ i & 0 \end{array}\right]$$

$$Pauli\;Z=\left[\begin{array}{cc} 1 & 0\\ 0 & -1 \end{array}\right]$$

$$Hadamard=\frac{1}{\sqrt{2}}\left[\begin{array}{cc} 1 & 1\\ 1 & -1 \end{array}\right]$$

(3) $$S=\left[\begin{array}{cc} 1 & 0\\ 0 & i \end{array}\right]$$

Such one-qubit gate can be used to form two-qubit gates, e.g., CNOT gate (accountable for the entanglement of qubits).

In order for QC to solve real world problems through quantum machine learning algorithms, classical data represented as multi-dimensional arrays need to be encoded, i.e., loaded into quantum systems as quantum states. This presents challenging open research because of the existence of various encoding methods [13,14] such as basis, angle, amplitude, etc., upon its usage is dependent on the structure of the dataset [15]. For instance, one embedding technique uses binary data to prepare a quantum state, another encodes the data into amplitudes of the quantum system or encodes the data into the rotation angles of the quantum gates. In general, each feature of the input data $x{\left(x_{0},x_{1},\ldots ,x_{n-1}\right)}^{T}\epsilon \;R^{n}$ is mapped to a quantum state $x\to \left|\left.\phi (x)\right\rangle \right.$ by applying an encoding circuit $U_{\phi }\left(x\right)$ acting on a ground state $\left|\left.0^{n}\right\rangle \right.$ of a Hilbert Space F described as $U_{\phi }\left(x\right)\left|\left.0^{n}\right\rangle \right.=\left|\left.\phi (x)\right\rangle \right.$ [16]. After this, the quantum data are sent through a quantum circuit W(θ), a series of quantum unitary operations, acting on $\left|\left.\phi (x)\right\rangle \right.$. The measurement phase is the decoding phase and decodes the quantum data through measurement into a classical form. In general, the Pauli-Z matrix is used as measurement method.

### 2.2. Classical Artificial Neural Networks

Image-processing tasks in machine learning can be categorized into supervised and unsupervised learning approaches. In supervised learning, a predetermined set of labeled training data, $X_{T}$, is used to generate corresponding classifications, $y\left(y_{0},y_{1},\ldots ,y_{n-1}\right)$, where $y_{i}$ represents the classification of $x_{i}$. The machine learning algorithm adjusts its internal parameters iteratively to achieve accurate classification on the training set y. Once the model has learned from the labeled data, it can be applied to classify new, unlabeled data instances denoted as X.

In contrast, unsupervised learning operates on unlabeled data, without predefined classifications. It aims to discover patterns and structures within the data without prior examples or knowledge. Clustering, a popular unsupervised learning technique, identifies subgroups or clusters within the dataset based on inherent similarities or patterns [17].

Artificial Neural Networks (ANNs) is a machine learning model. ANNs are inspired by functions of the human brain and describes a computational processing approach consisting of an input layer, hidden layer, and output layer. In the input layer, the data are loaded in the form of a multidimensional vector that gets sent to the hidden layers. The hidden layers are responsible for the learning where the computation is performed. Here, a weighted sum and bias is applied to the inputs and directed through an activation function to increase the non-linearity in the output. The process of learning takes place by making decision of previous layers and determining how a stochastic change improves the final output [18]. Moreover, ANNs with multiple deeper layers are referred to as deep neural network or deep learning. Deep learning models manipulate large datasets.

Convolutional Neural Networks (CNN) are deep neural networks that perform linear operations between matrices. In general, CNN consist of a convolutional layer (to detect meaningful features), non-linearity layer (saturates the output), pooling layer (reduces the complexity for further layers), and fully connected layer (enables the connectivity with all neurons in the preceding and succeeding layer) [19].

### 2.3. Variational Quantum Circuit (VQC)

Variational Quantum Circuits (VQC), also referred to as parameterized quantum circuits (PQC), are unitary operations performed on n qubits denoted as U_θ_, applied to an initial state |ϕ_0_⟩. The outcome results in a parameterized state |ψ_0_⟩ = U_θ_ |ϕ_0_⟩, where θ represents a set of tunable parameters [20]. This approach is known as the Variational Quantum Algorithm (VQA). In essence, the VQC acts as an interface between quantum and classical computers, where the expectation value is measured on the quantum computer, while the classical computer calculates the cost function C_θ_ for updating the parameters using an optimization problem. The objective is to find the circuit parameters θ for the PQC U_θ_ that minimize the cost function C_θ_. Furthermore, VQAs are regarded as Quantum Neural Networks (QNN) as they train the model (ansatz) by adjusting gate parameters to approximate an optimal solution for achieving accurate predictions. Furthermore, several variants of QNN have been proposed such as hybrid quantum-classical convolutional neural network (QCCNN) in Liu et al. [21], quanvolutional neural networks (QNNs) in Henderson et al. [22] and quantum convolutional neural network (QCNN) in Cong et al. [23] to process classical information into and out of the different quantum circuits to accurately determine the decision boundary of a classification problem.

## 3. Related Work

Various works of Breast Cancer Detection using classical as well as quantum machine learning method have been published in the last few years. In this article, we will focus on related work inspired by quantum neural network using data in the form of Breast X-ray, ultrasound images, and CT scans.

Mishra et al. [24] proposed a five-qubit quantum neural network architecture to detect breast cancer using qubits as neurons where the input features of the dataset are implemented as initial rotations on each qubit. Parameterized rotations of R_Z_ and R_X_ gates act on the neurons which are being optimized during training to achieve the minimum deviation from the obtained value from the true value. Furthermore, partial connection of neurons is achieved through entanglement using CX gates for which the technique of dropout known as in classical CNN is achieved. The model was trained on breast cancer MRI dataset of 600 samples using a logarithmic loss function with an average loss of 2.1%. Azevedo et al. [25] trained a hybrid classical–quantum neural networks using transfer learning to classify full-image mammograms into malignant and benign where a pre-trained ResNet18 serves as a feature extractor for a quantum classifier. The quantum classifier consists of a data embedding part using Hadamard (H) and R_y_ rotation gates and of a variational layer, also known as trainable circuit using entangling unitary operation made of three CNOT gates. The output of the variational layer is passed to classical linear layer to produce the output state. The proposed model achieved an accuracy of 84% against 76.9% to state-of-the-art solutions. Another approach is presented in the work of Amin et al. [26] where in the first phase breast lesions from histopathological and ultrasound images were segmented using a semantic segmentation consisting of a jointly model namely deepLabv3 and a pretrained Xception model. The method reached a global accuracy of benign and malignant classes of 0.999 and 0.998. The F-scores of benign and malignant classes were 0.993 and 0.990. In the second phase (influenced by the approach described in [22]), the authors converted the segmented images into a quantum image using a non-trainable quantum convolutional layer of a 4-qubit quantum circuit with $R_{y}$ rotation gates for the data embedding and 10 layers of randomly chosen single qubit rotations and 2-qubit entangling gates. The convolved images were then sent to a trainable six-layered classifier of four dense layers with one drop-out and flatten layers to classify the two classes benign and malignant. The proposed model was trained on the binary classification dataset (162 histopathological sliding photographs of breast cancer and 780 breast ultrasound images) and achieved 97% accuracy in classifying histopathological images and 99% on ultrasound images. In [27], the authors use hybrid quantum–classical convolutional neural networks (QCCNN) with different encoding methods and circuit architectures to classify radiological images to examine possible advantages of quantum machine learning in medical imaging analysis. The QCCNN models were compared to a classical CNN model with the result that QCCNN models require less trainable parameters to achieve similar performance as the classical ones.

In this study, unlike previous proposed methods, we have designed a quantum convolutional neural network with a fixed quantum circuit consisting of 9 qubits, which acts as a 3 × 3 quantum kernel filter to extract features from ultrasound breast images of female subjects.

## 4. Proposed Methodology

The proposed method consists of a pre-processing, data encoding, and a breast cancer detection phase where quantum convolutional kernels are used to create a feature map to outline detected features in the input image whose features are in turn sent to one fully connected layer (FC), also known as linear/dense layer for the final output classification.

### 4.1. Data Description and Pre-Processing

**Figure 1:**
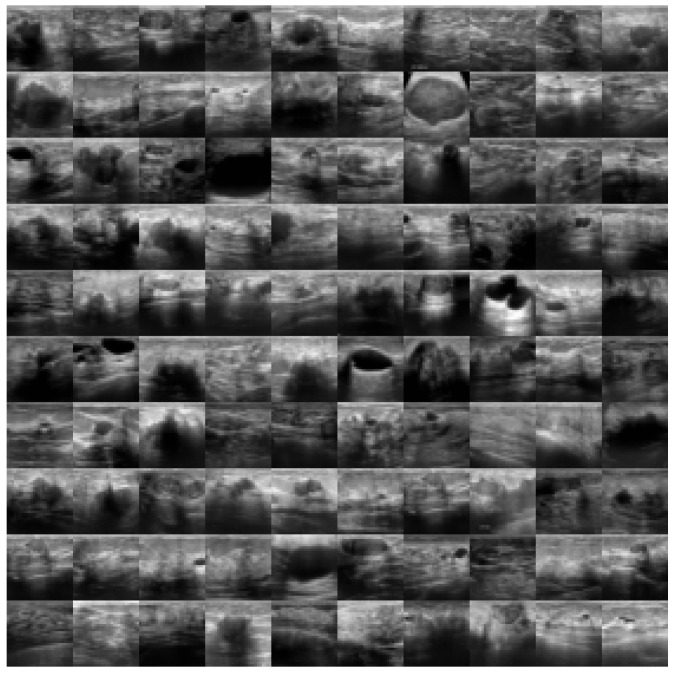
A 10 × 10 montage of samples from ultrasound breast images extracted from the training set, encompassing three distinct classes: normal, benign, and malignant, from the BreastMNIST dataset.

The dataset used in this experiment is BreastMNIST, a collection of 780 ultrasound breast images, as depicted in Figure 1. BreastMNIST encompasses three distinct classes: normal, benign, and malignant. Due to the utilization of low-resolution images, the classification task is simplified to binary classification. Here, normal and benign cases are amalgamated into the positive class, juxtaposed against the malignant class designated as negative.

In preparing BreastMNIST, the source dataset is portioned into training, validation, and test sets with a ratio of 7:1:2, respectively. Furthermore, the original images, each sized 1 × 500 × 500, undergo a resizing process to dimensions of 1 × 28 × 28 to suit the model architecture and computational requirements [28].

For the research, we created a nearly balanced dataset for the training phase by using a subset of the total training data. Subsequently, we downsized the original images to 14 × 14 pixels to reduce the training duration. Additionally, we normalized the input features to a range between 0 and 1 to ensure that the data are on a similar scale, thereby achieving better numerical stability and facilitating faster model convergence.

### 4.2. Architecture of the Quantum Classifier

**Figure 2:**
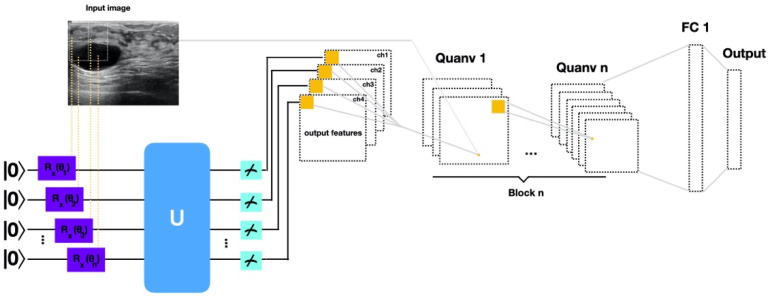
Illustration of the proposed method to use a quantum convolutional layer to generate a feature map. First, a 3 × 3 square matrix traverse across the image with a stride of 2 until it has swept across the entire image. The kernel filter is applied by embedding 3 × 3 pixels into the quantum system using angle embedding in the feature-embedding circuit. The encoded data are then sent through a quantum convolutional layer which works similarly to a classical convolutional layer. Quantum filters consisting of parameterized U3, entangling CNOT, and parameterized R_y_ and R_z_ gates are acting on the data. The final output, i.e., feature map is decoded with a measurement in the Pauli-Z basis. Subsequently, the output is flattened and sent through fully connected layers whereas the very last layer classifies the output.

The classifier consists of a quantum data embedding, quantum convolution part, measurement, and a classical dense layer as illustrated in Figure 2.

First, the classical data are embedded into quantum states using angle embedding with rotation angles X. Angle embedding encodes N normalized data points into the intervals $\left[0,\pi /2\right]$ into the rotation angles of *n* qubits, where N≤n. Here, the rotation is around the *x*-axis of the Bloch Sphere, and the angle of rotation is conditional on the input feature. The unitary for angle encoding is described as:

$$S_{x_{j}}={⨂}_{i=1}^{N}U\left(x_{j}^{\left(i\right)}\right)$$

where

(4) $$U\left(x_{j}^{\left(i\right)}\right)=R_{x}\left(2x_{j}^{\left(i\right)}\right)=\left[\begin{array}{cc} \cos\left(x_{j}^{\left(i\right)}\right) & -\sin\left(x_{j}^{\left(i\right)}\right)\\ \sin\left(x_{j}^{\left(i\right)}\right) & \cos\left(x_{j}^{\left(i\right)}\right) \end{array}\right]$$

What’s more, angle encoding operates on one data point at a time and maintains a constant quantum circuit depth, making it suitable for current hardware limitations [13,14].

In our proposed method, reminiscent of the strategy used in Henderson et al. [22], we encode a small region of the input image (here a 3 × 3 square, i.e., 9 qubits) sequentially. This encoded data is then processed through a quantum convolutional layer, consisting of a series of parameterized gates.

**Figure 3:**
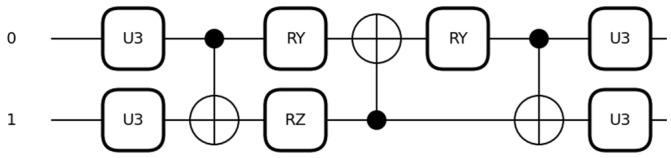
Quantum convolutional kernel is a parameterization of an arbitrary single-qubit gate U3 followed by CNOT, R_y_ and R_z_ gates.

The circuit for this quantum convolutional layer, which we employ in our method, consists of a strongly entangled circuit as it permits the classifier to attain different levels of correlations in the image data. The circuit in Figure 3 is a quantum convolutional kernel parameterized by an arbitrary single-qubit gate U3 followed by CNOT, R_y_, and R_z_ gates. Figure 3 illustrates the parameterization of an arbitrary SU(4) gate first introduced in [29], modified in [30], and used in our method as referenced in [31].

The circuit consists of single qubit gates U3 with three parameters θ, ϕ, and λ:

U3 = $\left[\begin{array}{cc} \cos\left(\theta /2\right) & {-e}^{i\lambda }\sin\left(\theta /2\right)\\ e^{i\phi }\sin\left(\theta /2\right) & e^{i\left(\lambda +\phi \right)}\cos\left(\theta /2\right) \end{array}\right]$, $R_{y}\left(\theta \right)=\left[\begin{array}{cc} cos(\theta /2) & -sin(\theta /2)\\ sin(\theta /2) & cos(\theta /2) \end{array}\right]$, $R_{z}\left(\phi \right)=\left[\begin{array}{cc} e^{-i\phi /2} & 0\\ 0 & e^{i\phi /2} \end{array}\right]$, and

(5) $$\text{two-qubit}\;\mathrm{CNOT}\;\mathrm{gates}\;\mathrm{CNOT}=\left[\begin{array}{c} 1\\ 0\\ 0 \end{array}\begin{array}{c} 0\\ 1\\ 0 \end{array}\begin{array}{c} 0\\ 0\\ 1 \end{array}\begin{array}{c} 0\\ 1\\ 0 \end{array}\right].$$

We adjusted the circuit and extended it to a 9-qubit circuit where U3 gate is applied to each qubit with CNOT gates applied between pairs of qubits, with one qubit acting as the control and the other as the target. R_y_ gate is applied before each CNOT gate on the first qubit of each pair whereas R_z_ between the first CNOT gates on the second qubit of each pair. To be precise, the chosen filter size of a 3 × 3 kernel size requires a 9-qubit circuit with 15 learnable parameters to produce 9 output feature maps. Here, the model uses two quantum convolutional layers.

As elucidated by Henderson et al. [22] alongside Havlicek at al. [32], quantum supremacy can only be achieved and thus outperform classical classification approaches with a belief rooted in two key factors. Firstly, quantum circuits are inherently hard to simulate using classical computational methods. Secondly, classical data are mapped using a quantum feature map into the Hilbert space of the quantum system. In this space, complex relationships and structures can be captured, and it can reveal non-linear relationships that may be challenging to represent using classical feature methods. Further, different from Henderson et al. [22] our approach uses angle encoding for data mapping and only quantum circuits with a predetermined, fixed sequence of gates and operations as convolutional layer. Furthermore, while we transform spatially local subsections of the input data through quantum convolutional filters, which allows us to train on large, high-dimensional datasets as also described in [22], we choose to train our model on a subset of the dataset. This approach is driven by our hypothesis that QNNs have the potential to perform on a small dataset. By doing so, we aim to address the challenge of training relatively small datasets, a common occurrence in medical imaging. In general, a small region of the classical input image, a 3 × 3 square, is embedded into the rotation angles of quantum rotation gates. Subsequently, quantum computation is applied to the system using variational quantum circuits serving as convolutional filters. The quantum system is then measured, producing a list of nine classical expectation values. These values are mapped into nine different channels of an individual output pixel. The procedure is applied over different regions using a stride of two until the entire image is scanned. The output is fed into another variational circuit. Next, the output is fed into a classical fully connected layer, which finally classifies the input into the desired labels using the sigmoid function. If the output is greater than the threshold of 0.5, then it is classified as positive (benign); otherwise, it is classified as negative (malignant).

## 5. Results

We used Pennylane 0.37.0, a python library for quantum machine learning algorithms [33], and Pytorch 2.3.1+cu121 [34] to conduct our experiment. We used Pennylane’s default_qubit device to perform a simple state simulation of qubit-based quantum circuit. Measurement results were obtained by calculating the expectation values of the PauliZ observables on wires. As performance measures, we employed Binary Cross Entropy (BCE) loss with logits and accuracy on both the training and validation datasets. The neural network was trained for 15 epochs using the Adam optimizer with a learning rate of 1 × 10^−2^ weight decay of 1 × 10^−5^ and a batch size of 10.

**Figure 4:**
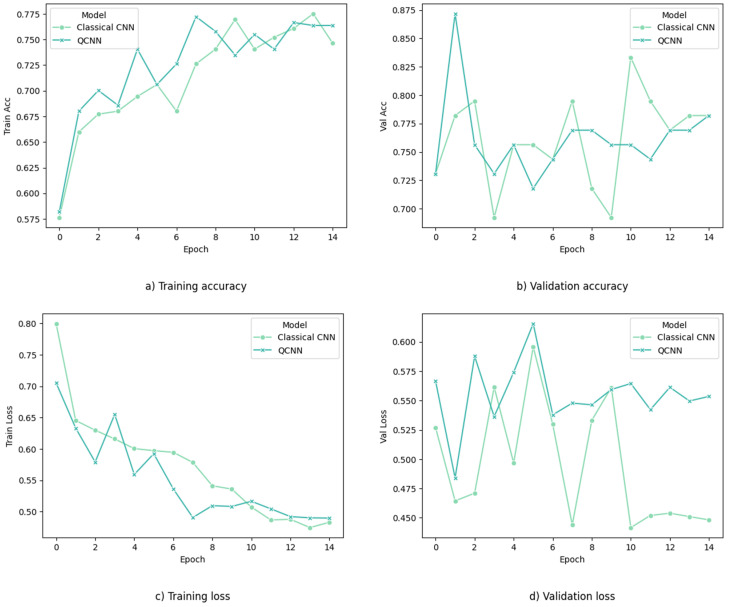
Performance of QCCN and classical CNN model with regard to training and validation accuracy and binary cross entropy with logits loss. QCNN achieves a peak training accuracy of 76.66% and validation accuracy 87.1%, whereas the classical CNN achieved an accuracy of 77.52% in training and 83.33% in validation.

To provide a benchmark for comparison, we implemented a classical model with an equivalent architecture consisting of the same number of convolutional and fully connected layers. Notably, the results demonstrate that our QCNN achieves a peak training accuracy of 76.66% and a validation accuracy of 87.17% with a learning rate of 1 × 10^−2^ using two quantum circuits as convolutional layers. The classical CNN model achieves a training accuracy of 77.52% and a validation accuracy of 83.33%, as shown in Figure 4. Subsequently, we tested the performance of the model on the test dataset using classification matrices.

The classifier performs better in identifying normal/benign cases (1) compared to malignant cases (0). Further, the precision for normal/benign cases is high (0.82), meaning that when it predicts benign, it is correct 82% of the time. The recall for malignant cases is 0.57, indicating that 57% of actual malignant cases are correctly identified. The overall accuracy is 67%, and the weighted averages reflect a slight preference towards the majority class (normal/benign).

Regarding Receiver Operating Characteristic (ROC); for Class 0 (malignant), TPR is 0.57, indicating a moderate ability to detect malignant cases while for Class 1 (normal, benign), TPR is 0.70, indicating a good ability to detect benign cases. Further, for Class 0 (malignant), FPR is 0.16, showing a relatively low rate of false positives while for Class 1 (normal, benign), FPR is 0.81, showing a high rate of false positives for malignant cases (Table 1).

This concludes that quantum circuits as convolutional layers can be solely used as kernel filters to extract features in images for classification models with a small dataset but improving the model’s performance might involve further investigation of topics such as data embedding techniques, gathering more data, or fine-tuning the model parameters to better distinguish between malignant and benign cases.

## 6. Discussion

In this study, we explored the use of quanvolutional neural networks as feature extractors for image classification using 3 × 3 feature kernel and a fixed circuit gate, aiming to understand their efficacy compared to classical convolutional neural networks (CNN). Our approach demonstrated that QCNNs could be employed effectively solely for feature extraction, differing from previous methods where quantum and classical layers alternated.

The performance of the quanvolutional neural networks was assessed using BreastMNIST with Binary Cross Entropy (BCE) loss with logits and accuracy metrics on the dataset. The model was trained for 15 epochs using the Adam optimizer with a learning rate of 1 × 10^−2^, weight decay of 1 × 10^−5^, and a batch size of 10. The model achieved a peak training accuracy of 76.66% and a validation accuracy of 87.17%, while the classical CNN model reached 77.52% and 83.33%, respectively. These results indicate that quanvolutional neural networks can perform competitively with classical models in certain aspects.

Training the quanvolutional neural networks presents several challenges, including the need for precise quantum operations, sensitivity to noise, and hardware imperfections. Quantum gates must be finely tuned, and even minor deviations can lead to substantial performance degradation. Additionally, the current state of quantum hardware often imposes limitations on the number of qubits and the depth of quantum circuits, restricting the complexity of the models that can be trained. The inherent stochastic nature of quantum measurements also adds an extra layer of variability, making the training process less stable compared to classical neural networks.

The ROC analysis and test performance highlighted that the model classifier performed better in identifying normal/benign cases (precision of 0.82) compared to malignant cases (recall of 0.57). The overall accuracy was 67%, with weighted averages showing a preference towards the majority class (normal/benign). The True Positive Rate (TPR) for malignant cases was moderate at 0.57, and good for benign cases at 0.70. The False Positive Rate (FPR) for malignant cases was low at 0.16, but high for benign cases at 0.81, indicating a higher rate of false positives for malignant cases.

These findings suggest that while quanvolutional neural networks can be effective for feature extraction, there are significant areas for improvement. The variability in quantum operations and hardware limitations need addressing to enhance performance stability and accuracy.

## 7. Conclusions

This study concludes that quantum circuits can be effectively used as convolutional layers to extract features in image classification models, even with small datasets. However, improving the model’s performance might involve utilizing such as advanced data embedding techniques, gathering larger datasets, and fine-tuning the model parameters to better distinguish between malignant and benign cases. Future work should focus on these aspects to enhance the efficacy and stability of quanvolutional neural networks.

## Figures and Tables

**Table 1:** Classification Metrics test dataset with a threshold of 0.5.

	Precision	Recall	f1-Score	Support
0	0.41	0.57	0.48	42
1	0.82	0.70	0.75	114
accuracy			0.67	156
macro avg	0.62	0.64	0.62	156
weighted avg	0.71	0.67	0.68	156
TPR	0.57	0.70		
FPR	0.16	0.81		

## Data Availability

The original contributions presented in the study are included in the article, further inquiries can be directed to the corresponding author.

## References

[1] Dromain C., Boyer B., Ferré R., Canale S., Delaloge S., Balleyguier C. (2013). Computed-aided diagnosis (CAD) in the detection of breast cancer. Eur. J. Radiol..

[2] Madani M., Behzadi M.M., Nabavi S. (2022). The Role of Deep Learning in Advancing Breast Cancer Detection Using Different Imaging Modalities: A Systematic Review. Cancers.

[3] Cruz J.A., Wishart D.S. (2006). Applications of Machine Learning in Cancer Prediction and Prognosis. Cancer Inform..

[4] Shen L., Margolies L.R., Rothstein J.H., Fluder E., McBride R., Sieh W. (2019). Deep Learning to Improve Breast Cancer Detection on Screening Mammography. Sci. Rep..

[5] Zeebaree D.Q., Haron H., Abdulazeez A.M., Zebari D.A. Machine learning and Region Growing for Breast Cancer Segmentation. Proceedings of the 2019 International Conference on Advanced Science and Engineering (ICOASE).

[6] Araújo T., Aresta G., Castro E., Rouco J., Aguiar P., Eloy C., Polónia A., Campilho A. (2017). Classification of breast cancer histology images using Convolutional Neural Networks. PLoS ONE.

[7] Solenov D., Brieler J., Scherrer J.F. (2018). The Potential of Quantum Computing and Machine Learning to Advance Clinical Research and Change the Practice of Medicine. Mo. Med..

[8] Sagingalieva A., Kordzanganeh M., Kenbayev N., Kosichkina D., Tomashuk T., Melnikov A. (2023). Hybrid Quantum Neural Network for Drug Response Prediction. Cancers.

[9] Vashisth S., Dhall I., Aggarwal G. (2021). Design and analysis of quantum powered support vector machines for malignant breast cancer diagnosis. J. Intell. Syst..

[10] Jain S., Ziauddin J., Leonchyk P., Yenkanchi S., Geraci J. (2020). Quantum and classical machine learning for the classification of non-small-cell lung cancer patients. SN Appl. Sci..

[11] Steane A. (1998). Quantum computing. Rep. Prog. Phys..

[12] Nielsen M.A., Chuang I.L. (2001). Quantum computation and quantum information. Phys. Today.

[13] LaRose R., Coyle B. (2020). Robust data encodings for quantum classifiers. Phys. Rev. A.

[14] Weigold M., Barzen J., Leymann F., Salm M. (2021). Encoding patterns for quantum algorithms. IET Quantum Commun..

[15] Abohashima Z., Elhosen M., Houssein E.H., Mohamed W.M. (2020). Classification with quantum machine learning: A survey. arXiv.

[16] Schuld M., Killoran N. (2018). Quantum Machine Learning in Feature Hilbert Spaces. Phys. Rev. Lett..

[17] Adcock J., Allen E., Day M., Frick S., Hinchliff J., Johnson M., Morley-Short S., Pallister S., Price A., Stanisic S. (2015). Advances in quantum machine learning. arXiv.

[18] O’Shea K., Nash R. (2015). An introduction to convolutional neural networks. arXiv.

[19] Albawi S., Mohammed T.A., Al-Zawi S. Understanding of a convolutional neural network. Proceedings of the 2017 International Conference on Engineering and Technology (ICET).

[20] Sim S., Johnson P.D., Aspuru-Guzik A. (2019). Expressibility and Entangling Capability of Parameterized Quantum Circuits for Hybrid Quantum-Classical Algorithms. Adv. Quantum Technol..

[21] Liu J., Lim K.H., Wood K.L., Huang W., Guo C., Huang H.-L. (2021). Hybrid quantum-classical convolutional neural networks. Sci. China Phys. Mech. Astron..

[22] Henderson M., Shakya S., Pradhan S., Cook T. (2020). Quanvolutional neural networks: Powering image recognition with quantum circuits. Quantum Mach. Intell..

[23] Cong I., Choi S., Lukin M.D. (2019). Quantum convolutional neural networks. Nat. Phys..

[24] Mishra N., Bisarya A., Kumar S., Behera B.K., Mukhopadhyay S., Panigrahi P.K. (2019). Cancer Detection Using Quantum Neural Networks: A Demonstration on a Quantum Computer. arXiv.

[25] Azevedo V., Silva C., Dutra I. (2022). Quantum transfer learning for breast cancer detection. Quantum Mach. Intell..

[26] Amin J., Sharif M., Fernandes S.L., Wang H., Saba T., Khan A.R. (2022). Breast microscopic cancer segmentation and classification using unique 4-qubit-quantum model. Microsc. Res. Tech..

[27] Matic A., Monnet M., Lorenz J.M., Schachtner B., Messerer T. Quantum-classical convolutional neural networks in radiological image classification. Proceedings of the 2022 IEEE International Conference on Quantum Computing and Engineering (QCE).

[28] Yang J., Shi R., Ni B. Medmnist classification decathlon: A lightweight automl benchmark for medical image analysis. Proceedings of the 2021 IEEE 18th International Symposium on Biomedical Imaging (ISBI).

[29] MacCormack I., Delaney C., Galda A., Aggarwal N., Narang P. (2022). Branching quantum convolutional neural networks. Phys. Rev. Res..

[30] Hur T., Kim L., Park D.K. (2022). Quantum convolutional neural network for classical data classification. Quantum Mach. Intell..

[31] Schuld M., Bocharov A., Svore K.M., Wiebe N. (2020). Circuit-centric quantum classifiers. Phys. Rev. A.

[32] Havlíček V., Córcoles A.D., Temme K., Harrow A.W., Kandala A., Chow J.M., Gambetta J.M. (2019). Supervised learning with quantum-enhanced feature spaces. Nature.

[33] Bergholm V., Izaac J., Schuld M., Gogolin C., Ahmed S., Ajith V., Killoran N. (2018). Pennylane: Automatic differentiation of hybrid quantum-classical computations. arXiv.

[34] Paszke A., Gross S., Massa F., Lerer A., Bradbury J., Chanan G., Chintala S. Pytorch: An imperative style, high-performance deep learning library. Proceedings of the 33rd Annual Conference on Neural Information Processing Systems.

